# Spatial accessibility to HIV testing, treatment, and prevention services in Illinois and Chicago, USA

**DOI:** 10.1371/journal.pone.0270404

**Published:** 2022-07-27

**Authors:** Jeon-Young Kang, Bita Fayaz Farkhad, Man-pui Sally Chan, Alexander Michels, Dolores Albarracin, Shaowen Wang

**Affiliations:** 1 Department of Geography Education, Kongju National University, Gongju-si, Chungcheongnam-do, South Korea; 2 Annenberg School for Communication, University of Pennsylvania, Philadelphia, Pennsylvania, United States of America; 3 CyberGIS Center for Advanced Digital and Spatial Studies, University of Illinois Urbana-Champaign, Urbana, Illinois, United States of America; 4 Illinois informatics Institute, University of Illinois Urbana-Champaign, Urbana, Illinois, United States of America; 5 University of Pennsylvania, Philadelphia, Pennsylvania, United States of America; 6 Department of Geography and Geographic Information Science, University of Illinois Urbana-Champaign, Urbana, Illinois, United States of America; SUNY Downstate Health Sciences University, UNITED STATES

## Abstract

Accomplishing the goals outlined in “Ending the HIV (Human Immunodeficiency Virus) Epidemic: A Plan for America Initiative” will require properly estimating and increasing access to HIV testing, treatment, and prevention services. In this research, a computational spatial method for estimating access was applied to measure distance to services from all points of a city or state while considering the size of the population in need for services as well as both driving and public transportation. Specifically, this study employed the enhanced two-step floating catchment area (E2SFCA) method to measure spatial accessibility to HIV testing, treatment (i.e., Ryan White HIV/AIDS program), and prevention (i.e., Pre-Exposure Prophylaxis [PrEP]) services. The method considered the spatial location of MSM (Men Who have Sex with Men), PLWH (People Living with HIV), and the general adult population 15–64 depending on what HIV services the U.S. Centers for Disease Control (CDC) recommends for each group. The study delineated service- and population-specific accessibility maps, demonstrating the method’s utility by analyzing data corresponding to the city of Chicago and the state of Illinois. Findings indicated health disparities in the south and the northwest of Chicago and particular areas in Illinois, as well as unique health disparities for public transportation compared to driving. The methodology details and computer code are shared for use in research and public policy.

## 1. Introduction

In 2019, the US Department of Health and Human Services (HHS) launched “Ending the HIV Epidemic: A Plan for America Initiative,” an agenda that seeks to reduce the number of new HIV (Human Immunodeficiency Virus) infections by 75% in the next five years (2025) and by 90% in the next ten years (2030). The initiative rests on deploying critical strategies, including diagnosing HIV as early as possible after infection, treating people living with HIV (PLWH) to achieve viral suppression, and preventing new HIV transmissions by using effective interventions (e.g., pre-exposure prophylaxis [PrEP]) to reduce HIV risk. Currently, HIV screening is recommended regularly for at risk groups such as Men Who Have Sex with Men (MSM) and at least once for 13 to 64 year old age groups [1]. Treatment is indicated for PLWH as early as possible following diagnosis, and prevention with PrEP (Pre-Exposure Prophylaxis) is recommended for groups at risk, including MSM [2]. Therefore, improving access to HIV testing, treatment, and prevention services for these populations is critical to realizing the goals of HIV eradication, making computational methods to estimate population access to these services vital.

Despite the importance of access to services, the evidence on patterns of access and the methods to gauge access are limited [3]. Access to health care is generally conceptualized as an interplay of different factors, such as availability of service providers, provider characteristics, affordability of services, and accommodation based on hours of operation. However, the local availability of service sites is the primary requirement for people to receive appropriate HIV services [4]. Thus, mapping service sites within a city or state is important to determine spatial accessibility, but appropriate mapping of spatial accessibility involves taking into account the balance between supply (i.e., the number of service sites) and demand (i.e., the number of people who seek a service) within the particular city or state [5]. Therefore, understanding spatial access to services involves simultaneously geolocating available service sites and the number of inhabitants who will need to use each service within the geographic area being considered. For example, establishing the spatial accessibility of PrEP requires overlapping a service map of PrEP sites with a population map of MSM.

Prior studies have mainly used three methods to measure spatial accessibility to services. The first method involves measuring travel time/distance to services. Across health domains, longer time to travel to healthcare services is associated with worse health outcomes (e.g., survival rates, length of stay in hospital), as reported in over 70% of the studies included in a systematic review [6]. In the HIV context, physical proximity to services determines usage of HIV care services [7], and the nearest service provider from the perspective of geographic distance is typically the most likely provider [8]. Moreover, people are less likely to be screened for HIV when the testing sites are more distant [9]. However, calculating the distance or travel time between the locations of residence and a clinic is not a complete assessment of service accessibility. In particular, proximity does not guarantee the availability of particular services because the number of people who need the healthcare service in the area is important as well [10]. For example, accessibility is likely to be poor if there are few service providers within a short distance but a large population in need of the service.

The second method estimates a supply access score per service site based on the characteristics of patients and service providers in a particular region. The supply access scores can then be compared with the target population to identify underserved areas [4,11–13]. The third method focuses on analyzing geographic accessibility by estimating the spatial distributions and the capacity of available healthcare services [11]. It combines locations of HIV care sites with population-weighted travel time and uses a threshold to identify suboptimal spatial accessibility to HIV care. In the US, the threshold is more than 30 minutes, a duration at which a service site is deemed inaccessible.

Past research on HIV has involved measuring spatial accessibility to HIV care for PLWH [3]. Ganapati et al. examined having a Ryan White HIV/AIDS care provider within 5 miles of the residence of PLWH as a marker of high accessibility. Although these estimates valuable, as explained before, not weighing accessibility by the size of the population of PLWH is problematic. Second, to the best of our knowledge, there are no prior analyses of spatial accessibility to other HIV services such as PrEP providing sites, or estimates of access for specific populations, such MSM. Third, spatial accessibility to services is impacted by different transportation modes [14,15]. For example, even though low-income people may be more likely to access HIV services by public transportation rather than by driving, the impact of transportation modes on spatial accessibility to HIV services has not been well studied.

To estimate spatial accessibility to HIV testing, treatment, and prevention services, we applied the enhanced two-step floating catchment (E2SFCA) method to analyze the spatial accessibility of treatment services, prevention services, and testing services, respectively for three populations: (a) PLWH, (b) MSM, and (c) people between the ages of 15 to 64. Multiple extensions of the two-step floating catchment area (2SFCA) method [16] have been developed, including for example, an enhanced two-step floating catchment (E2SFCA) method [17], a parallel-enhanced two-step floating catchment (PE2SFCA) method [18], and variable two-step floating catchment (V2SFCA) method [19]. Most relevant to our study, the accessibility measurement from the E2SFCA method can identify places or regions where health care resources are relatively abundant or scarce, normalized by the size of the population (see Section 3 for detail). Despite its advantages, to the best of our knowledge, the 2SFCA method has not been utilized to assess HIV care.

In summary, this study aims to answer the following research questions: 1) How do we map spatial accessibility to HIV testing, treatment, and prevention services for PLWH, MSM, and people aged 15 to 64 in a city or state? 2) How does such spatial accessibility to HIV testing, treatment, and prevention services vary across space? 3) What is the impact of transportation mode (i.e., road network and public transportation) on spatial accessibility in each case? We analyzed the state of Illinois and the city of Chicago, where spatial accessibility has been identified as partly responsible for the increased risk of HIV transmission among Illinois residents [20]. The results can identify which regions have relatively high (vs. low) spatial accessibility to HIV testing, treatment, and prevention services, indicating which regions may need additional investment in public health infrastructure.

## 2. Materials and methods

### 2.1. Study area and data

#### HIV services

**Fig 1:**
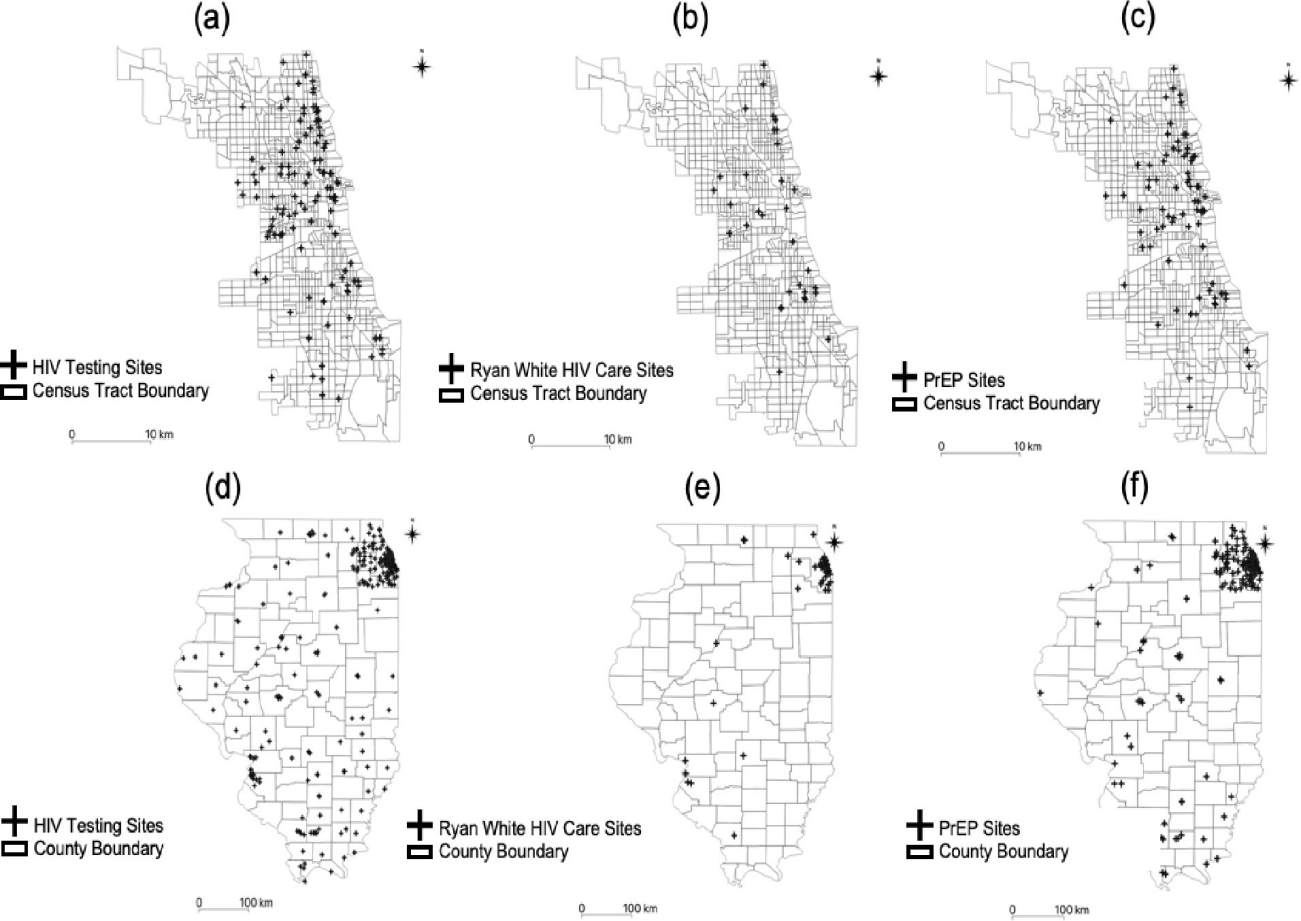
Distributions of HIV testing, treatment, and prevention sites. (a) HIV Testing Sites in Chicago; (b) Ryan White HIV Care Sites in Chicago; (c) PrEP Sites in Chicago; (d) HIV Testing Sites in Illinois; (e) Ryan White HIV Care Sites in Illinois; and (f) PrEP Sites in Illinois.

We created a dataset of HIV services, including testing, treatment, and prevention, located in Illinois and Chicago using the Locator.HIV.gov website (https://locator.aids.gov). The Locator.HIV.gov website provides information about each service site, including name, address, phone number, and available services (i.e., HIV testing, sexually transmitted infection [STI] testing, PrEP, Ryan White HIV/AIDS care, health center, mental health, substance abuse, housing assistance, and family planning). We recorded the name, the address of available HIV testing, treatment (i.e., Ryan White HIV/AIDS Program), and prevention sites (e.g., PrEP clinics). Approximately 1,100 HIV service sites were available in Illinois, and about 600 of them provided testing, treatment, and prevention services (Fig 1). We assumed that all service sites have the same service capacity, which means that each facility can serve the same number of people.

#### Target populations

**Fig 2:**
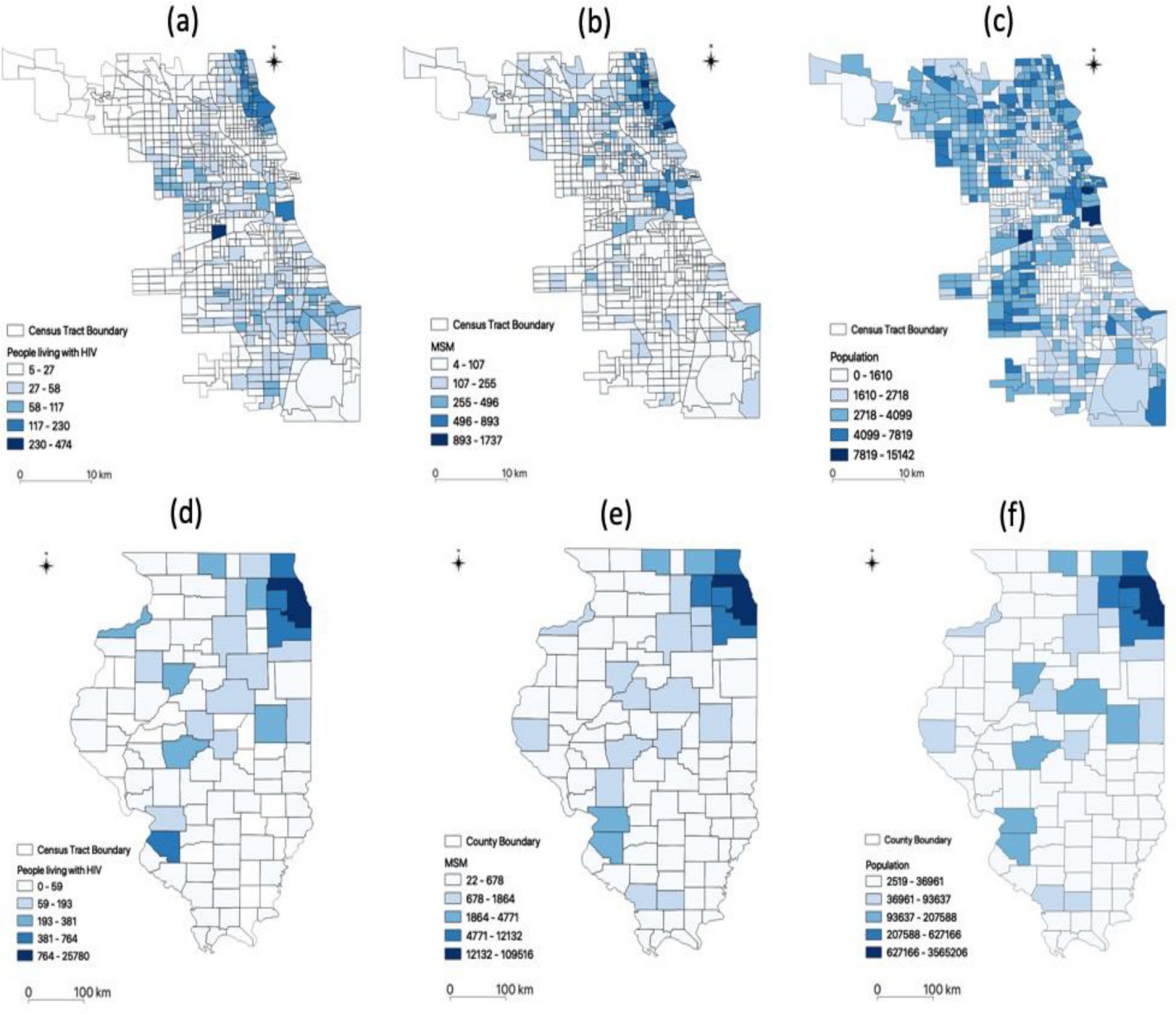
Three types of population in Chicago and Illinois. (a) PLWH in Chicago; (b) population (aged 15 to 64) in Chicago; (c) MSM in Chicago; (d) PLWH in Illinois; (e) general population (aged 15 to 64) in Illinois; and (f) MSM in Illinois.

We examined three types of populations: (1) PLWH, (2) MSM, and (3) people aged 15 to 64 (Fig 2). We used county-level datasets of PLWH, MSM estimates from the year 2017, and people aged 15 to 64. AIDSVu (https://map.aidsvu.org/map) provides data on PLWH in Chicago at the census tract level for the year 2017 and in the State of Illinois at the county level for the year 2016. The American Community Survey (ACS) provided the number of people aged 15 to 64 at the census tract level for the year 2017 and at the county level for the year 2016. The ACS provides annually updated information on demographic and socio-economic characteristics of people and housing in the U.S. The census tract is contained within a county, and each tract contains a population of approximately 4,000 people. We followed the equations and the percentages of same-sex male (SSM) households in urban-rural areas published in Grey, Bernstein [21] to estimate the size of the MSM population in census tracks and counties. The details of MSM estimates appear in the Appendix.

#### Road network and public transportation

**Fig 3:**
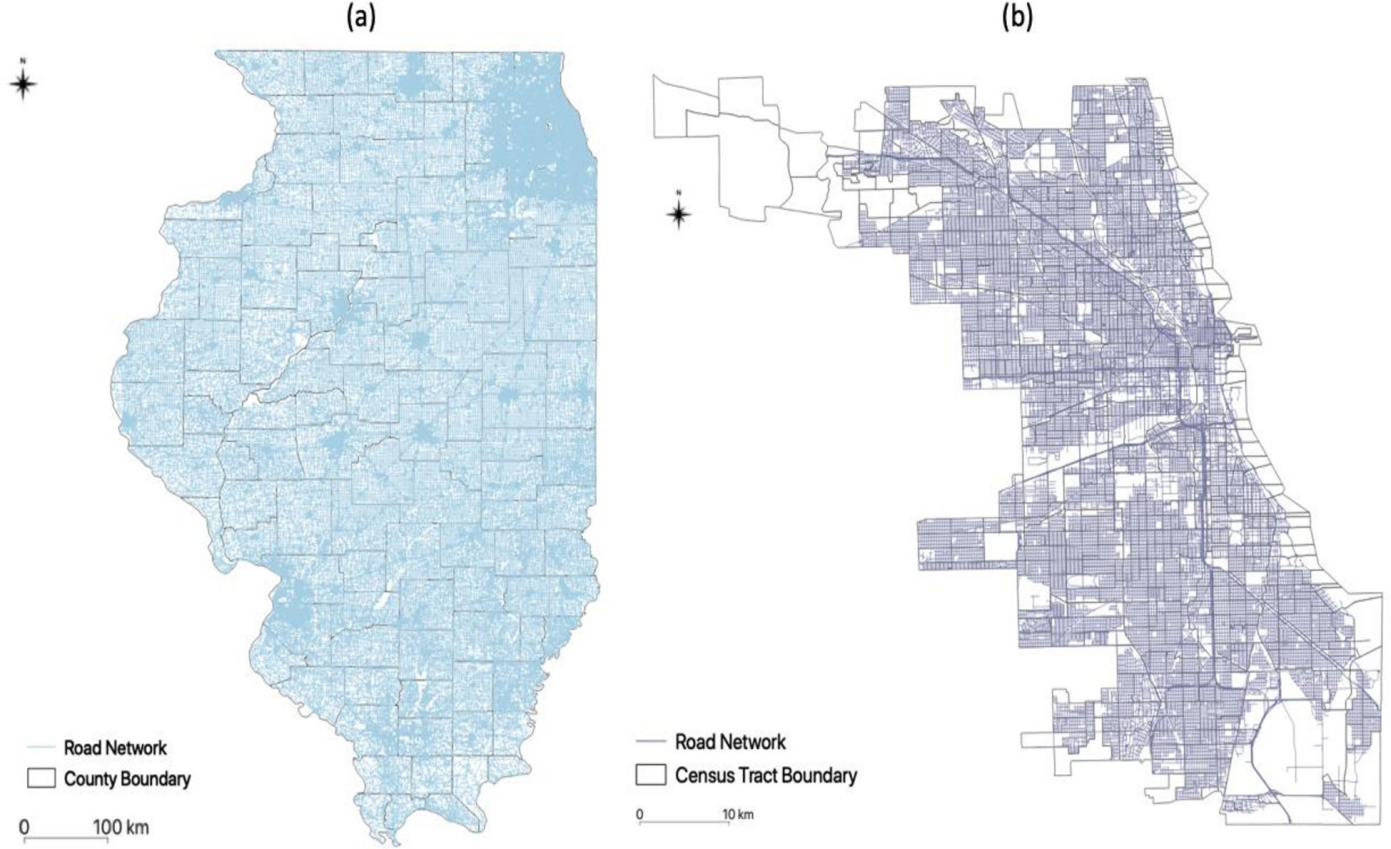
Road network: (a) Illinois and (b) Chicago.

We obtained a transportation network with approximately 334,343,000 segments from the OpenStreetMap Python library (OSMnx), which allows for the acquisition, construction, and analysis of a road network in specific regions [22]. We next used the road network data to calculate travel distance for each population and to identify the catchment area of each HIV service site. Fig 3 illustrates the road network in (a) Illinois and (b) Chicago. The O’Hare International Airport is located in the northwest of Chicago where no road network data is available. Thus, we excluded this area in our analyses. In addition to the road network data, we also collected public transportation data in Chicago, which is provided by Google as part of the General Transit Feed Specification (GTFS). The GTFS data provide public transit information as a type of routable network graph with stops as nodes, and trips between stops as edges.

### 2.2. An enhanced two-step floating catchment area (E2SFCA) method

This study used the enhanced two-step floating catchment area (E2SFCA) method [17] to measure the spatial accessibility of HIV services. The E2SFCA method improves a similar method, called the two-step floating catchment area (2SFCA) method, by considering the effects of distance decay within a catchment area by setting multiple travel time zones, such as 0–10, 10–20, and 20–30 minutes [16].

The supply-to-population ratio *R*_*j*_ is computed at a location *j* as:

$$R_{j}=\frac{S_{j}}{\sum_{k\in \{d_{kj}\leq d_{0}\}}P_{k}}$$ (1)

A catchment area of each service site (*j*) is delineated based on a threshold distance (*d*_*0*_). If the population centroid (*k*) falls within a catchment area, the sum of the population at *k* is the denominator in the Eq (1). Then, *S*_*j*_ denotes the number of available HIV testing, treatment, and prevention services (*S*) at each site (*j*). Because we focused on the availability of the services, the equation does not consider the number of resources (e.g., physicians or beds) per site. Therefore, a value of 1 is applied into *S*_*j*_. Importantly, the population here refers to the number of PLWH, people aged 15 to 64, and MSM.

**Fig 4:**
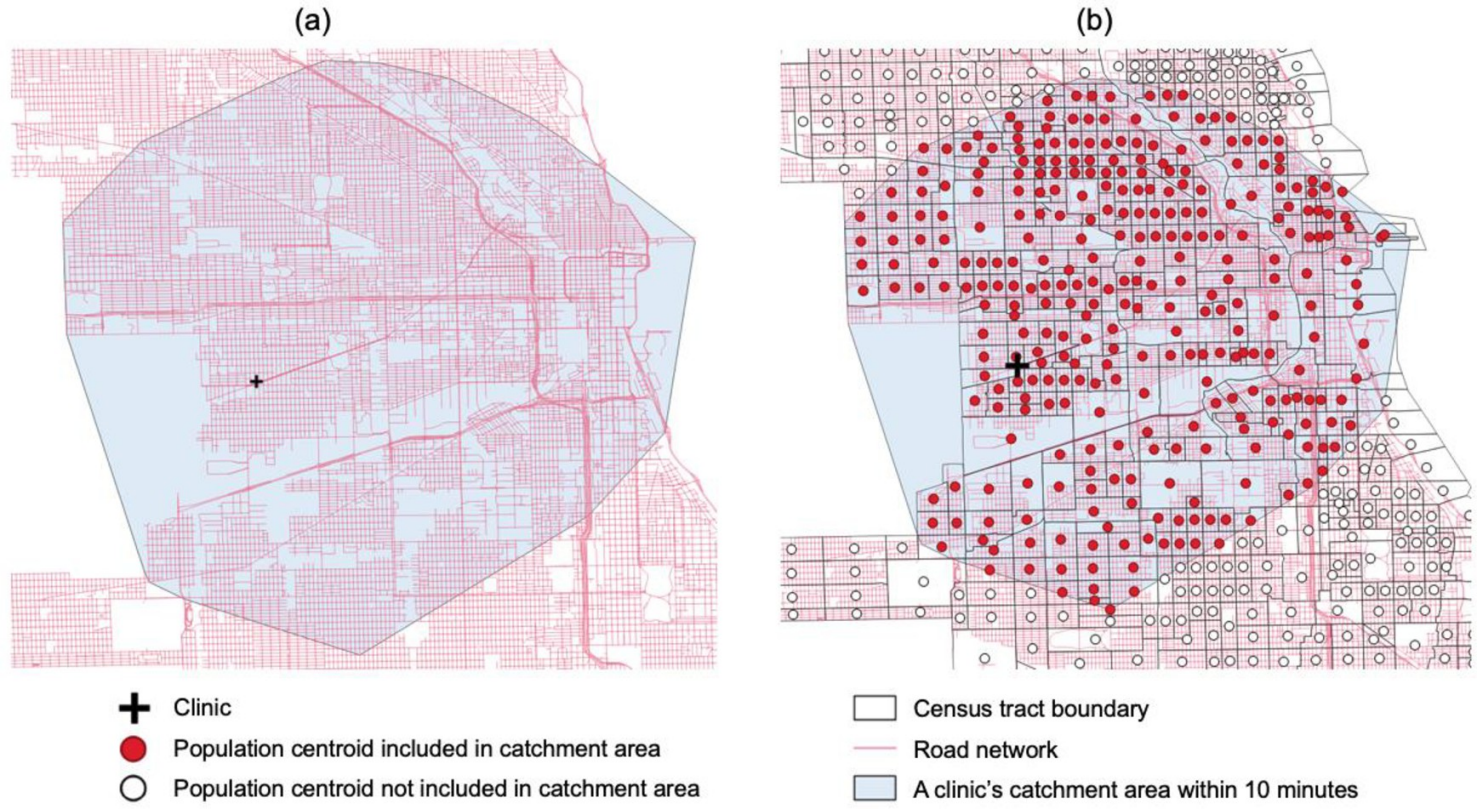
Delineate a clinic’s catchment area: (a) a clinic’s catchment area; (b) population centroids included in the catchment area.

Fig 4 illustrates how to delineate the catchment area per service site and how to measure a clinic’s spatial accessibility. The use of road network enables researchers to identify the nodes reaching to the travel time (e.g., 10, 20, and 30 minutes) from the service site. Then, a convex hull polygon can be constructed, which delineates a service site’s catchment area (Fig 4A), and overlapping population centroids with the convex hull are identified (Fig 4B). If the population centroids fall within the service site’s catchment area, the sum of the population serves as a denominator in the Eq (1).

We used the GTFS dataset to delineate the catchment area of service sites in Chicago. It contains the locations of bus stops and stations, as well as transfer locations in Chicago. We applied the shortest-path algorithm [23] to compute the shortest path between the closest transit stop from each HIV testing, treatment, and prevention service location and that from each population’s centroid. Then, we used road segments of public transportation with a constant transit speed of 10 miles per hour [24] to estimate the travel time.

Spatial accessibility to specific types of HIV services is defined as the summation of the scores of the spatial accessibility per service site in a region. It also takes into consideration travel zones, such as 0–10, 10–20, and 20–30 minutes. The values of 1, 0.68, and 0.22 are used as weights for travel zones 0–10, 10–20, and 20–30 mins, respectively [17].

$$A_{i}=\sum_{k\in (d_{ij}\in D_{1})}R_{j}W_{1}+\sum_{k\in (d_{ij}\in D_{2})}R_{j}W_{2}+\sum_{k\in (d_{ij}\in D_{3})}R_{j}W_{3}$$ (2)

where *A_i_* denotes the accessibility of people at location *i* to sites, and the proportions of physicians-to-population *R_j_* at service sites *j* whose centroids are located within the catchment area. *W_r_* denotes the distance weight for *r*th travel intervals. We repeated the E2SFCA analyses multiple times to estimate spatial accessibility to testing, treatment (i.e., Ryan White HIV/AIDS Program), and prevention (i.e., PrEP) services. Spatial accessibility to HIV testing was analyzed for MSM and people aged 15 to 64; access to treatment (i.e., The Ryan White HIV/AIDS Program) was analyzed for PLWH. Spatial accessibility to prevention (i.e., PrEP) was analyzed for the general population aged 15 to 64 and for MSM.

**Fig 5:**
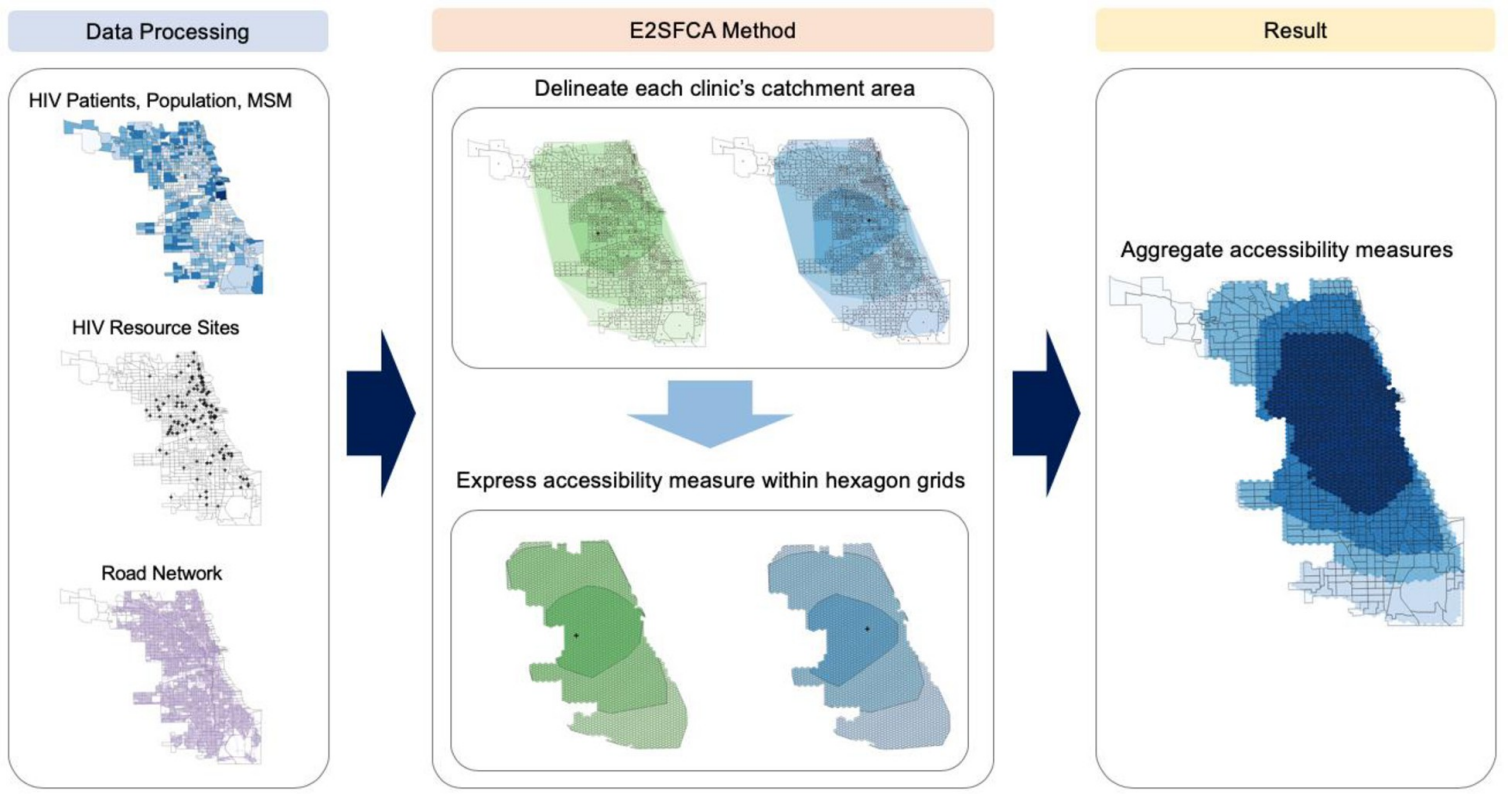
Workflow. Note: The value of each hexagon is determined by *A*_*i*_ from Eq (2).

Fig 5 illustrates the analytical procedures. We first collected datasets about population (i.e., PLWH, people aged 15 to 64, MSM), HIV service sites, and the road network. Next, we applied the E2SFCA method to measure spatial accessibility by delineating each site’s catchment area based on travel distance zones (i.e., 0–10, 10–20, and 20–30 minutes), depicting within hexagon grids to minimize the orientation bias from edge effects, and aggregating the scores of all service sites within a region into hexagon grids. Even though in Chicago, the shortest distance between census track centroids is about 130 meters, such a fine resolution may be responsible for preventing effective visualization. Therefore, for Chicago, we used 500-meter hexagon grids. For Illinois, we used 5-km hexagon grids, which are more appropriate to represent vast rural areas within the state.

The E2SFCA method is computationally intensive when handling large road networks and aggregated accessibility measures. Therefore, we used cyberGIS (i.e., cyber geographic information science and systems) to resolve such computational intensity [25–27]. In addition, to enable computational reproducibility, our analysis workflow has been made openly available as a CyberGIS-Jupyter notebook [28,29].

## 3. Results

We first report the results of spatial accessibility to HIV testing, treatment, and prevention services using the road network data and then the patterns of spatial accessibility among HIV services in the context of Illinois and Chicago. Following that presentation, we describe the results of the impact of transportation modes on spatial accessibility to services in Chicago using the road network and GTFS data, respectively.

### 3.1. Illinois

**Fig 6:**
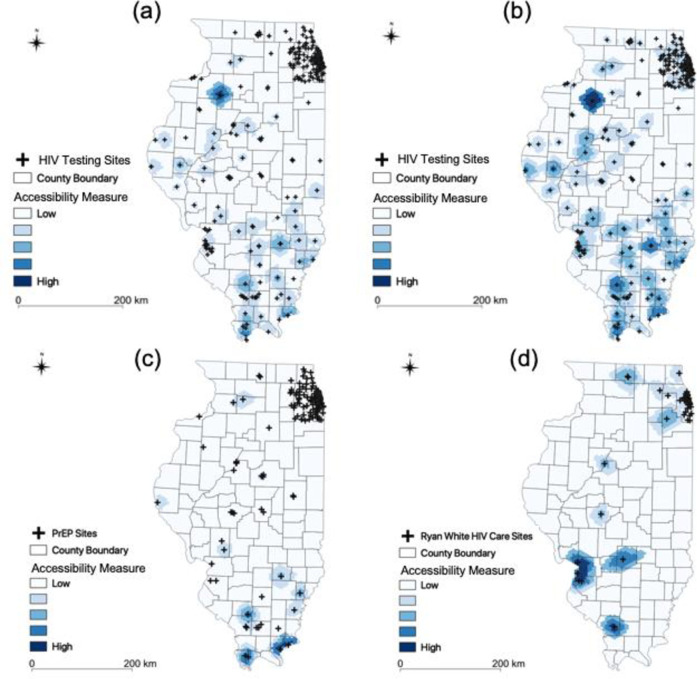
Spatial accessibility in Illinois. (a) Testing for MSM; (d) Testing for people aged 15 to 64; (c) Prevention for MSM; (d) Treatment for PLWH.

We investigated spatial accessibility to HIV testing, treatment, and prevention services for our target populations in Illinois. We found different levels of spatial accessibility for different regions, populations, and HIV services, and the results appear in Fig 6. With respect to regions and services, the results showed different levels of spatial accessibility across regions in Illinois. Also, as shown, there are large areas with low accessibility for all three services and no area had adequate spatial accessibility to all three services. Moreover, many areas had high spatial accessibility to HIV testing but low spatial accessibility to both treatment and prevention services.

With respect to populations and services, for MSM and people aged 15 to 64, Kewanee had the highest level of accessibility to HIV testing, followed by Mound and Rosiclare. Second, for MSM and prevention, Rosiclare had the highest level of accessibility, followed by Pulaski, Du Quoin, and Flora. Third, for PLWH, the St. Louis area had the highest level of accessibility to the Ryan White HIV/AIDS Program, followed by Carbondale, Vandalia, Cherry Valley, and Rockdale.

We next performed the Kruskal-Wallis test and the post-hoc Dunn’s test with Bonferroni adjustment to examine whether differences in spatial accessibility of services were statistically significant. As shown in Table 1, there were significant differences in the accessibility measures, as indicated by the confidence intervals for the normalized special accessibility and high levels of heterogeneity across the board (*H = 389*.*92*, *p<0*.*001*). The results from the Dunn’s tests can be used to compare normalized spatial accessibility and showed that (a) the levels of accessibility to testing for MSM and 15 to 64 year old age groups were higher than the level of accessibility to prevention for MSM (*p*<0.001); (b) the level of accessibility to treatment was higher than the level of accessibility of prevention (*p*<0.001); and (c) the level of accessibility to testing was higher than the level of accessibility to treatment (*p*<0.001). Thus, more resources need to be invested to increase the accessibility of both prevention and treatment than that of testing.

**Table 1:** Spatial accessibility of HIV testing, treatment, and prevention services in Illinois.

Service	Normalized Spatial Accessibility (95% CI)
HIV Testing for MSM	0.0325 (0.0308–0.0342)
HIV Testing for people 15 to 64	0.0328 (0.0312–0.0344)
HIV Prevention for MSM	0.0157 (0.0144–0.0170)
HIV Treatment for PLWH	0.0198 (0.0180–0.0216)

### 3.2. Chicago

#### 3.2.1. Spatial accessibility by road network (driving)

**Fig 7:**
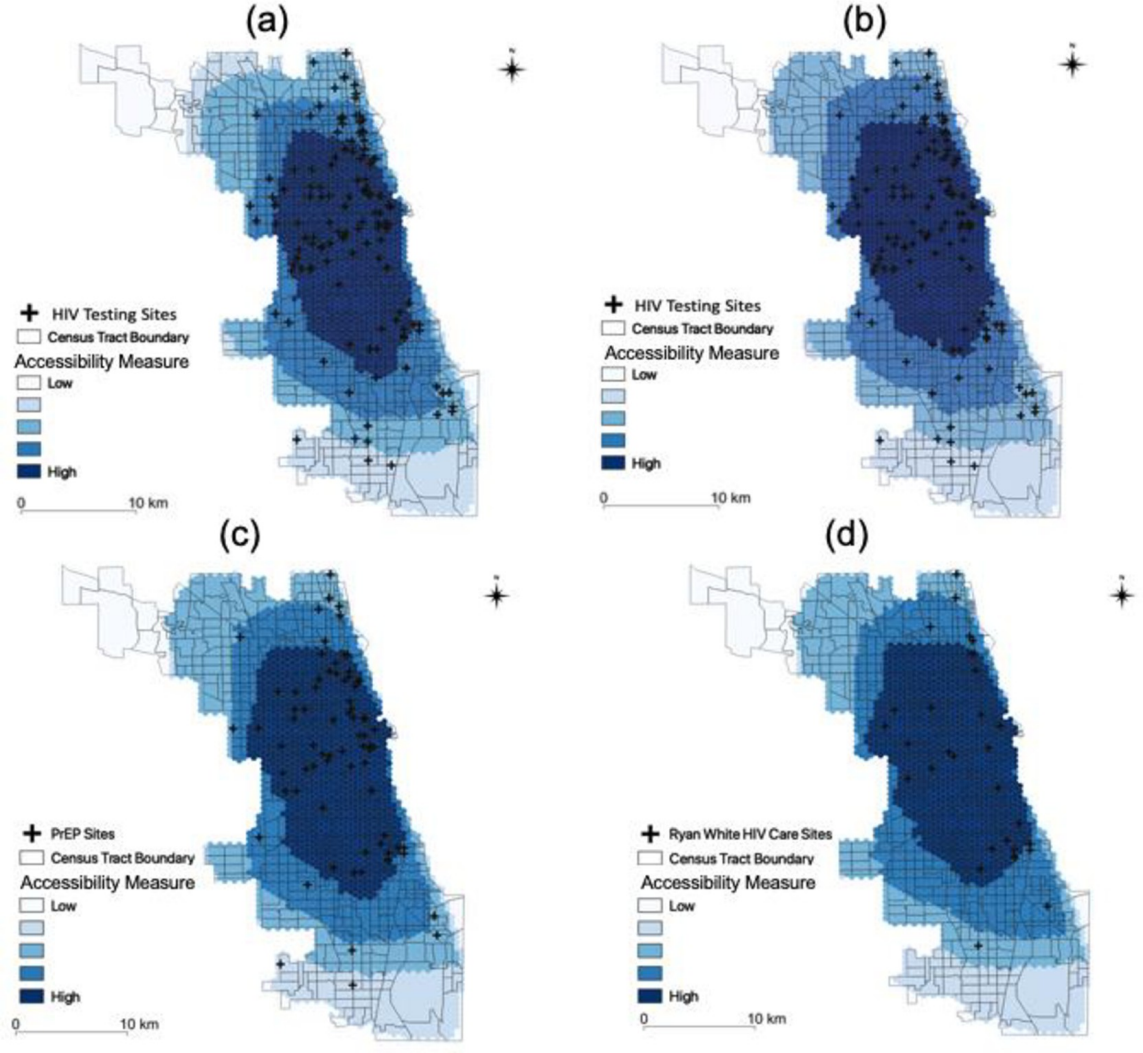
Spatial accessibility in Chicago. (a) Testing for MSM; (d) Testing for people aged 15 to 64; (c) Prevention for MSM; (d) Treatment for PLWH.

As with the state data, the results of Chicago also showed variable spatial accessibility of HIV testing, treatment, and prevention services (Fig 7). PLWH, MSM, and people aged 15 to 64 residing in the center of Chicago had higher spatial accessibility than those living in the northwest and south of Chicago. This pattern of spatial accessibility is largely due to most service sites being located in the center of the city.

The results of the Kruskal-Wallis test revealed statistically significant differences in accessibility (*H = 9*.*8955*, *p <0*.*05*). The confidence intervals for the normalized accessibility measure appear in Table 2. The results from the Dunn’s test also showed that the level of accessibility of treatment was lower than that the level of accessibility of testing for MSM (*p <0*.*05*).

**Table 2:** Accessibility measure in Chicago. (a) Treatment for PLWH; (b) Prevention for MSM; (c) Testing for MSM and people aged 15 to 64.

Service Type	Normalized Accessibility Measure (95% CI)
HIV Testing for MSM	0.6214 (0.6120–0.6308)
HIV Testing for people 15 to 64	0.6155 (0.6059–0.6252)
HIV Prevention for MSM	0.6118 (0.6024–0.6213)
HIV Treatment for PLWH	0.6044 (0.5949–0.6138)

#### 3.2.2. Spatial accessibility by public transport

**Fig 8:**
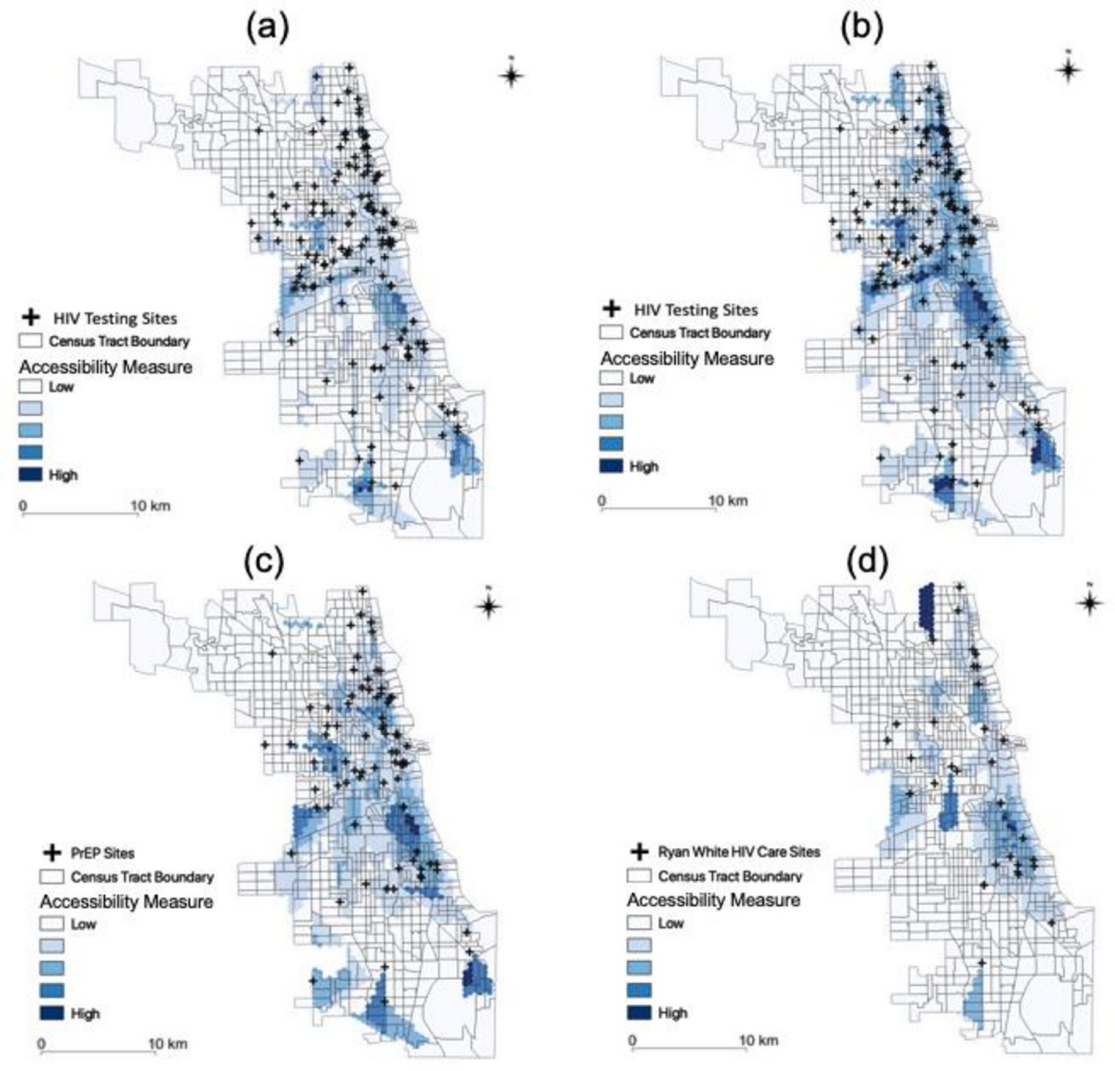
Accessibility measure in Chicago. (a) Testing for MSM; (d) Testing for people aged 15 to 64; (c) Prevention for MSM; (d) Treatment for PLWH.

Fig 8 shows distinct accessibility patterns for public transport. These patterns were different from the patterns identified using road network data, which assumes that a person drives to each service location (Fig 7). In general, the accessibility of HIV services was higher in downtown Chicago because of extensive public transportation networks in the area.

The results of Kruskal-Wallis test indicated high statistically heterogeneity in spatial accessibility (*H = 389*.*92*, *p <0*.*001*), which is displayed in Table 3. Moreover, the results from the Dunn’s test showed that (a) the level of accessibility to testing for people aged 15 to 64 was higher than the level of accessibility to prevention for MSM and testing for MSM (*p <0*.*05*); (b) the level of accessibility to treatment for PLWH was lower than the level of accessibility to prevention for MSM, to testing for MSM, and to testing for people aged 15 to 64 (*p < 0*.*05*).

**Table 3:** Accessibility measure in Chicago.

Service Type	Normalized Accessibility Measure (95% CI)
HIV Testing for MSM	0.0306 (0.0285–0.0327)
HIV Testing for people 15 to 64	0.0424 (0.0399–0.0448)
HIV Prevention for MSM	0.0481 (0.0448–0.0514)
HIV Treatment for PLWH	0.0461 (0.0417–0.0505)

To examine the impact of transportation mode on spatial accessibility in Chicago, we carried out Mann-Whitney tests because the distributions of driving and public transportation were not normal. The results showed statistically significant heterogeneity in the spatial accessibility of all HIV services between those by driving and public transits (*p <0*.*001*). Not surprisingly, spatial accessibility of all HIV services by driving was greater than that by public transportation.

## 4. Concluding discussion

Spatial access to HIV testing, prevention, and treatment cannot be assessed by only travel distance or time. In this study, we first investigated the spatial accessibility of HIV testing, prevention, and treatment services in Illinois. Using the well-known method of E2SFCA, we accurately measured spatial accessibility of those services in three populations, including PLWH, MSM, and population aged 15 to 64. Results from the E2SFCA method helped to identify which geographic areas require additional testing, prevention, and treatment services, considering the size of the population that needs each service. We found varied accessibility of each service across areas in Illinois. We also applied the method to Chicago using the road network and public transportation data to assess the impact of transportation mode.

From Kruskal-Wallis tests, we found that there are statistically significant differences in the level of accessibility to services in Chicago and in Illinois. HIV testing, treatment, and prevention services are not equally distributed across areas of Illinois or Chicago. At the state level, people who need HIV testing resources are more accessible than those who need either prevention or treatment resources. In addition, Mann-Whitney tests showed statistical differences between driving and public transportation. The results suggest that people living in Chicago have relatively lower accessibility to HIV testing, treatment, and prevention services than do other residents of Illinois. At the city level, when it comes to the spatial accessibility via driving, MSM and people 15 to 64 have better accessibility to HIV-related resources than PLWH. With respect to the accessibility via public transportation, the spatial accessibility to prevention resources among MSM and that to treatment among PLWH is relatively greater than the accessibility to testing resources for MSM and people 15 to 64. In addition, people living in the northwest of Chicago have low accessibility to HIV services regardless of whether spatial accessibility is assessed for driving or public transportation. These results imply a need to reconsider resource allocation to reduce spatial inequality of HIV care resources in Chicago and possibly other major U.S. cities as well. Although HIV services may be administratively prioritized based on larger administrative jurisdictions, improving accessibility means that service providers need to be located in the areas with higher service demands [3]. In this regard, our approach to characterizing spatial accessibility to HIV prevention highlights areas with limited access to care or prevention services and may inform the future allocation of resources to address existing disparities. For suburban and more densely populated rural areas, the provision of services through alternative venues (e.g., pharmacies and federally qualified healthcare centers) [30,31] and mobile vans [32,33]) should probably expand. For more remote, rural areas, telemedicine options may be optimal. Particularly, due to the COVID-19 outbreaks, the Health Resources and Services Administration’s (HRSA) Ryan White Program has encouraged PLWH to use telehealth to avoid traveling to seek care [34]. Therefore, telemedicine options are increasingly available and improving as well as expanding telemedicine practice hold great potential for mitigating inequalities in the spatial accessibility of services. Our results also suggest that accessibility to PrEP is the lowest among essential HIV testing, treatment, and prevention services. Enhancing spatial accessibility to PrEP is thus essential. In addition to our analysis, it is important to make our computational workflow openly available as a CyberGIS-Jupyter notebook as a way of achieving reproducibility. This open science practice not only allows our work to be applied to different geographic settings but also enables validation and verification of our results.

There are several limitations to our study. First, the accessibility measures of HIV testing, prevention, and treatment services may be underestimated because we did not consider that people who live near state boundaries may visit healthcare resources in neighboring states, including Indiana, Wisconsin, Iowa, Kentucky, and Missouri. Second, given the presence of low-income MSM in the south of Chicago [35], multiple transportation modes (e.g., bus, subway, bike, and walk) need to be taken into account [15,36]. Therefore, exploring the combined impacts of various transportation modes on the accessibility of HIV testing, treatment, and prevention services is worth future research. Third, we did not consider varying capacity of HIV services at each site, which is an issue for future research. Fourth, to improve understanding of temporally varying spatial accessibility, it would be important to measure spatial accessibility over time. Lastly, we did not consider race, ethnicity, health insurance status, and homelessness in our analyses. Given that, in the US, a large number of HIV infections is related to the socioeconomic, behavioral, clinical characteristics, and homelessness [37,38], examining their associations with the spatial accessibility to HIV-related resources is an important next step of our ongoing research.

Policymakers have announced a national commitment to end the HIV epidemic in the US. Particularly, in the state of Illinois, regular meetings among regional HIV strategy stakeholders have been held to achieve a more integrated and better coordinated response to the HIV epidemic [20]. The state response includes preventing new HIV infections, improving health outcomes for PLWH, reducing HIV-related health disparities, and avoiding HIV-related stigma and discrimination [20]. Our findings highlight the magnitude of health disparities in access to HIV testing, prevention, and treatment services. Given the well-documented link between travel time to health services and adverse health conditions, such spatial accessibility variations may exacerbate existing disparities in the overall HIV epidemic in the United States. This study may thus guide programs to increase the availability of HIV services in underserved areas of Illinois and the US.

## Supporting information

S1 Appendix (DOCX)
