# Supplementary material for: Spatial accessibility to HIV testing, treatment, and prevention services in Illinois and Chicago, USA
Source: PLoS One. 2022 Jul 27;17(7):e0270404. doi: 10.1371/journal.pone.0270404 (PMC9328561; doi:10.1371/journal.pone.0270404)
Supplement: S1 Appendix — (DOCX) [file pone.0270404.s001.docx]

**Appendix**

To estimate MSM population, we followed a method developed by Grey, Bernstein (21), which computes the MSM population based on the urbanicity-specific MSM prevalence and urbanicity-specific prevalence of male-male households. The MSM Prevalence is as follows: 4.4 % of adult males in large central metro, 2.5 % of adult males in large fringe metro, 1.4 % of adult males, and 1.1 % of adult males in non-metro region. The male-male household prevalence is in the following: 0.39 % of households in large central metro, 0.20 % of households in large fringe metro, and 0.20% of households in medium/small metro, and 0.11 % of households in non-metro region.

The urbanicity-specific MSM prevalence and male-male household prevalence are applied in the following equations.

1. Non-metropolitan/rural counties

$$\frac{(\% of all households in county that are male-male}{0.11\%}*1.1 \%*adult men in county$$

2. Medium/small metropolitan counties

$$\frac{(\% of all households in county that are male-male}{0.20\%}*1.4 \%*adult men in county$$

3. Large fringe metropolitan counties

$$\frac{(\% of all households in county that are male-male}{0.20\%}*2.5 \%*adult men in county$$

4. Large central metropolitan counties

$$\frac{(\% of all households in county that are male-male}{0.39\%}*4.4 \%*adult men in county$$

Figure A1 and Table A1 provide the level of urbanicity in Illinois [21].


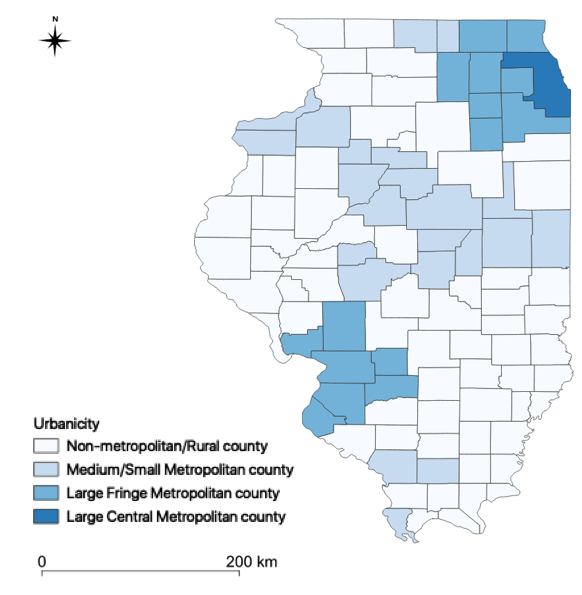


Figure A1. Urbanicity in Illinois

Table A1. Counties by the Level of Urbanicity

| Level of Urbanicity | Counties |
| --- | --- |
| Non-Metropolitan/Rural county | Adams, Brown, Cass, Lawrence, Hardin, Clark, Edgar, Shelby, Moultrie, Jasper, Effingham, Iroquois, Edwards, Wayne, Wabash, Cumberland, Douglas, Coles, Jefferson, Marion, White, Saline, Galltin, Hamilton, Randolph, Perry, Washington, Knox, Logan, Mson, Fulton, Warren, Schuyler, McDonough, Union, Henderson, Pulaski, Fayette, Massac, Johnson, Clay, Hancok, Montgomery, Pope, Crawford, Livingston, Morgan, Richland, Scott, Pike, Lee, Franklin, Christian, Putnam, Whiteside, Bureau, LaSalle, Greene, Carrol, Ogle, Stephenson, Jo Daviess, Kankakee, Calhoun |
| Medium/Fringe Metropolitan county | Henry, Rock Island, Mercer, Alexander, Williamson, Sangamon, Menard, Jackson, Champaign, Boone, Peoria, Vermilion, McLean, Winnebago, Piatt, De Witt, Tazewell, Stark, Woodford, Macon, Marshall, Ford |
| Large Fringe Metropolitan county | Monroe, DuPage, Gundy, Will, Kane, Madison, St. Clair, Clinton, Bond, McHenry, Dekalb, Lake, Kendall, Macoupin, Jersey |
| Large Central Metropolitan county | Cook |
